# Correction: Efficient photocatalytic performance of hydrogen bonding between P25 and microcrystalline cellulose aerogel

**DOI:** 10.1039/d5ra90100f

**Published:** 2025-09-01

**Authors:** Junhao Zeng, Di Zhang, Guoliang Dou, Qingming Zeng, Jian Zhang

**Affiliations:** a Shandong Engineering Research Center for High Performance Silicone Rubber Weifang 261000 P. R. China sduchemzhang@163.com; b Department of Chemistry and Chemical Engineering, Jining University Qufu 273100 P. R. China

## Abstract

Correction for “Efficient photocatalytic performance of hydrogen bonding between P25 and microcrystalline cellulose aerogel” by Junhao Zeng *et al.*, *RSC Adv.*, 2024, **14**, 37546–37553, https://doi.org/10.1039/D4RA07087A.

The authors regret an error in the spelling of Junhao Zeng’s name in the original article. The correct spelling is as shown in this Correction article.

The Royal Society of Chemistry apologises for these errors and any consequent inconvenience to authors and readers.

